# The functional interplay of low molecular weight thiols in *Mycobacterium tuberculosis*

**DOI:** 10.1186/s12929-018-0458-9

**Published:** 2018-07-12

**Authors:** C. Sao Emani, M. J. Williams, I. J. Wiid, B. Baker

**Affiliations:** DST-NRF Centre of Excellence for Biomedical Tuberculosis Research; SAMRC Centre for Tuberculosis Research; Division of Molecular Biology and Human Genetics; Department of Biomedical Sciences, Faculty of Medicine and Health Sciences; Stellenbosch University, PO Box 241, Francie van Zijl Drive, Tygerberg 8000, Cape Town, South Africa

**Keywords:** Thiols, Compensation, Tuberculosis, Therapeutic targets, ROS, RNS

## Abstract

**Background:**

Three low molecular weight thiols are synthesized by *Mycobacterium tuberculosis (M.tb)*, namely ergothioneine (ERG), mycothiol (MSH) and gamma-glutamylcysteine (GGC). They are able to counteract reactive oxygen species (ROS) and/or reactive nitrogen species (RNS). In addition, the production of ERG is elevated in the MSH-deficient *M.tb* mutant, while the production of MSH is elevated in the ERG-deficient mutants. Furthermore, the production of GGC is elevated in the MSH-deficient mutant and the ERG-deficient mutants. The propensity of one thiol to be elevated in the absence of the other prompted further investigations into their interplay in *M.tb*.

**Methods:**

To achieve that, we generated two *M.tb* mutants that are unable to produce ERG nor MSH but are able to produce a moderate (Δ*egtD-mshA*) or significantly high (Δ*egtB-mshA*) amount of GGC relative to the wild-type strain. In addition, we generated an *M.tb* mutant that is unable to produce GGC nor MSH but is able to produce a significantly low level of ERG (Δ*egtA-mshA*) relative to the wild-type strain. The susceptibilities of these mutants to various in vitro and ex vivo stress conditions were investigated and compared.

**Results:**

The Δ*egtA-mshA* mutant was the most susceptible to cellular stress relative to its parent single mutant strains (ΔegtA and ∆mshA) and the other double mutants. In addition, it displayed a growth-defect in vitro, in mouse and human macrophages suggesting; that the complete inhibition of ERG, MSH and GGC biosynthesis is deleterious for the growth of *M.tb*.

**Conclusions:**

This study indicates that ERG, MSH and GGC are able to compensate for each other to maximize the protection and ensure the fitness of *M.tb.* This study therefore suggests that the most effective strategy to target thiol biosynthesis for anti-tuberculosis drug development would be the simultaneous inhibition of the biosynthesis of ERG, MSH and GGC.

## Background

Upon host invasion, *Mycobacterium tuberculosis* (*M.tb*) encounters various cellular stresses generated by macrophages such as acidic, oxidative and nitrosative stress. However, *M.tb* is able to escape this, multiply, causing active tuberculosis (TB) or become dormant within necrotic macrophages (granuloma) [1, 2]. The host susceptibility to TB depends largely on its ability to fight invading mycobacteria by generating reactive oxygen species (ROS) and reactive nitrogen species (RNS) [3]. This became evident when the NADPH oxidase deficient (NOX2) mice and iNOS (inducible nitric oxide synthase) deficient mice were found to be more susceptible to TB infection than the wild-type mice [4, 5]. In addition, children with chronic granulomatous disease (a disorder characterized by phagocytic oxidative bursts resulting in recurrent pyogenic infections) were found to be highly susceptible to TB and to present complications during BCG vaccination [6]. Previous studies suggest a role of the phagocyte NADPH oxidase in the release of cytokines, implicating an interplay between the production of cytokines and the production of ROS, though the mechanism remains ambiguous. In addition, this interplay may also be implicated in the structural organization and formation of granuloma [7].

Several enzymes have been implicated in the detoxification of *M.tb* [8–10], however these enzymes required redox buffers during enzymatic reactions. Low molecular weight thiols such as MSH and ERG are efficient redox buffers [8, 11, 12]. Though it was shown that MSH and ERG are required for the survival of mycobacteria during adverse conditions [11, 13–15], the ability to generate mycobacteria mutants deficient in either MSH or ERG or both [11, 13, 16–18], suggests that they are compensated for by another thiol. Recently, another thiol, gamma-glutamylcysteine (GGC), was shown to provide mycobacteria protection against nitrosative and oxidative stress [18]. In addition, the production of ERG is elevated in the absence of MSH and vice versa [11, 15–19], while the production of GGC was found to be elevated in a MSH-deficient mutant and ERG-deficient mutants [18]. Elevation of one thiol following the loss of another as a compensation mechanism to maximize the protection of *M.tb* was therefore investigated in this study*.* Double mutants lacking MSH and ERG but producing a moderate amount of GGC (Δ*egtD-mshA*), another lacking both MSH and ERG but producing a high level of GGC (Δ*egtB-mshA*) and a last one lacking both MSH and GGC but producing a low level of ERG (Δ*egtA-mshA*) were generated. These were further characterized in vitro and ex vivo. This study demonstrates for the first time the significance of the compensatory roles of ERG, MSH and GGC in *M.tb.*

## Methods

### Generation and genotyping of *M.tb* double mutants

The gene *mshA* was deleted in the previously generated single *M.tb* CDC1551 mutants (*egtA*, *egtB* and *egtD*) as previously described [18, 20, 21]. To maximize the chances of obtaining the double mutants, plates were supplemented with OADC and ERG during the double cross over step. The deletion of *mshA* was investigated by PCR as previously described [18] and confirmed by southern blotting as follows.The genomic DNA was extracted from each strain as previously described [22]. Following extraction, it was digested (4–6 μg) with ClaI restriction enzyme overnight. Confirmation of complete digestion was performed by running a little amount of the digested DNA on an agarose gel (test gel). The digested genomic DNA along with the digoxigenin (DIG)-labelled molecular weight ladder (Roche) were separated on a 1% agarose gel and depurinated by incubating the gel in 250 mM HCl for 10 min (mins) at room temperature (RT). The gel was later rinsed with distilled H_2_O.

### Southern transfer of DNA onto the membrane

This was performed as previously described [23] with few modifications. After depurination, the DNA was denatured by incubating the gel at room temperature (RT) for 30 mins in the denaturing buffer (0.5 M NaOH, 1.5 mM NaCl). It was subsequently neutralised in the neutralization buffer (0.5 M Tris/HCl pH 7.5, 1.5 M NaCl) for another 30 mins. Southern transfer was performed using the positively charged nylon membrane (Roche Diagnostic GmbH Mannheim, Germany) overnight in 20X SSC (3 M NaCl, 300 mM sodium citrate, pH 7). The membrane was washed in 2X SSC the following day and baked for 2 h (hrs) at 80 °C between 2 Wattman papers.

The DIG High Prime DNA labelling and detection kit II and the DIG wash and block Buffer set (Roche) were used at this stage. The principle is described briefly as follows. DIG binds to the T and A bases of the single stranded (denatured) probe, which would hybridize to the single stranded fragments from the genomic DNA digestion. Subsequently, an anti-DIG antibody (conjugated to alkaline phosphatase) would be added. The conjugated alkaline phosphatase would be able to dephosphorylate CSPD (Disodium3-(4-methoxyspiro{l,2-dioxetane-3,2′-(5′-chloro)tricyclo[3.3.1.13,7]decan}-4-yl) phenyl phosphate) leading to photons emission at 477 nm.

### Probe labelling

An amount of 150–200 ng of the probe DNA was denatured at 100 °C for 10 mins, snapped cooled on ice for 5 mins, added in 1 X DIG High prime and incubated for 24–48 h at 37 °C for the labelling reaction to occur. The reaction was stopped by incubating the mixture at 65 °C for 10 mins.

### Prehybridization and hybridization of the membrane

The membrane from the southern transfer, was pre-hybridized at 65 °C for 30–60 min in the pre-hybridization buffer using a shaking water bath (optimal hybridization temperature is equal to the melting temperature of the probes minus forty two). The denatured probe was added in the hybridization buffer and the pre-hybridization buffer was replaced with the hybridization buffer containing the probe. Using a heat sealer, the membrane was sealed without air bubbles in a plastic bag and incubated at 65 °C overnight. The following day, the membrane was washed twice for 5 mins at RT in a high stringency preheated (65 °C) buffer (2X SSC/0.1% SDS (sodium dodecyl sulphate)). It was subsequently washed twice in a preheated (65 °C) low stringency buffer (0.5X SSC/0.1% SDS) for 15 mins. After these washes, the membrane was incubated with shaking at RT for 2 mins in 1X maleate buffer. After discarding the maleate buffer, the membrane was incubated further in the blocking solution for 30 mins to 3 hrs. The blocking solution was discarded and replaced with another blocking solution containing the specific antibody and the membrane was incubated further for another 30 mins. The membrane was subsequently washed twice in 1X washing buffer at RT for 15 mins and equilibrated with the detection buffer for 3 mins.

### Detection

The equilibrated membrane was placed in a hybridization bag and 1-3 ml CSPD (chemiluminescent substrate for alkaline phosphatase) was applied to it. After removal of air bubbles, the membrane was heat-sealed. Following, incubation at RT for 5 mins, excess fluid was removed, and the membrane was sealed again and incubated further at 37 °C for 10 mins. Bands detection and image acquisition were performed by the ChemiDoc™ MP System (BioRad; 2000 Alfred Nobel Drive, Hercules, California 94,547, USA).

### Quantification of thiols

After culturing mycobacteria for ~ 2 weeks in 7H9 liquid media, 100 μl of each culture was serially diluted and plated for colony forming unit (CFU) counts. Five millilitres of each culture was pelleted by centrifugation. One millilitre of the supernatant was filtered twice (using a syringe filter) and lyophilized overnight. The pellets were washed twice in double distilled water and stored at − 80 °C. Lyophilized supernatants (extracellular fractions) and frozen pellets (intracellular fractions) from all biological replicates were re-suspended in the lysis buffer (50% warm acetonitrile + 20 mM hepes pH 8 + 2 mM monobromobimanne), sonicated in a water bath sonicator for ~ 30 mins at 60 °C and acidified with a final concentration of 1 mM acetic acid. Following centrifugation, they were filtered twice and stored for liquid chromatography tandem mass spectrometry (LC-MS) analyses [24].

### Growth curve analysis of the mutants

This was performed as previously described [25]. The growth rate was evaluated by subtracting the OD_600_ at day 4 from the OD_600_ at day 10, which represents the linear exponential phase of growth [26].

### Susceptibility testing of the mutants

Mycobacteria from frozen stocks were cultured on plates, 7H11 solid cultures supplemented with 1X OADC (oleic acid, albumin, dextrose and catalase). The lawn of mycobacteria formed on the plates were scrapped off, re-suspended in 7H9 supplemented with 1X ADS (albumin, dextrose, sodium chloride). After disrupting cell aggregates by trituration, cells suspensions were re-suspended to an OD_600_ of ~ 0.02. Then they were exposed in a 96-well plate to either 0.2 mM diamide (DIM), or 5 mM vitamin C (VC) for ~ 72 h, or ~ 4 mM diethylaminetriamine nitric oxide adduct (DETA-NO) for 14 days. Their susceptibilities to diamide (DIM) and vitamin C (VC) were estimated from the CFU counts of the treated cells relatively to the untreated cells. Their susceptibilities to DETA-NO were estimated from the fluorescence intensity of resazurin of the treated cells relative to the untreated cells. Similarly, their susceptibilities to antibiotics were investigated as follows. Logarithmic phase liquid cultures (in ADS) were diluted to an OD_600_ of ~ 0.007 and added to an equal volume of serially diluted drugs in black 96-well plates (optical bottom) and incubated for ~ 7 days at 37 °C. After adding resazurin and incubating the plates for 24–48 h at 37 °C, susceptibilities were estimated from the measured fluorescence intensity of resazurin (excitation at 544 nm and emission at 590 nm) of the treated cells relative to the untreated cells.

### Infection of macrophages

Double mutants were inoculated 3–7 days before other strains, to ensure that they are at the same growth stage on the day of infection. On the day of infection, logarithmic phase mycobacteria were washed in phosphate buffer saline (PBS) or in the defined cell media supplemented with 10% fetal bovine serum (RPMI 1640 (Roswell Park Memorial Institute medium) for human primary macrophages or DMEM (Dulbecco’s modified Eagle’s medium) for RAW 264.7 murine cell lines). Re-suspensions were triturated by mixing with a syringe (25GA × 5/8in (0.5 x16mm)) (≥10X). The OD_600_ was adjusted to 0.1 with the cell culture media and the re-suspension was triturated. Previous optimizations revealed that the concentration of the double mutants, *ΔegtA-mshA* and *ΔegtD-mshA* was ~ 10^7^/ml, of the *∆egtB* and ∆*egtB-mshA* mutants was ~ 4 X 10^7^ and of the other mutants and the wild-type was ~ 3.5 X 10^7^/ml at OD_600_ of ~ 0.1. This enabled to calculate the volume required for each strain to obtain the targeted multiplicity of infection (MOI). One ml of each adjusted suspension was added in triplicate and accordingly to each well of a 24-well plate containing seeded macrophages (~ 5 X 10^5^ per well). Few microliters of the mycobacterial suspensions added to the macrophages were serially diluted and plated for CFU counts to estimate the number of mycobacteria at time zero (before infection). Macrophages were gently washed (two-four times) after three hours of infection. Following the wash step, they were either lysed with 0.05% SDS in order to estimate the intracellular mycobacteria load at time ~ 4 h or incubated further for later time points. For prolong exposure, additional washes were performed on the third day. M0 macrophages differentiated from peripheral blood mononuclear cells (PBMC) isolated over the Histopaque (Sigma Aldrich) gradient method from the blood of a healthy donor were infected as described above with the only difference in the number of macrophages seeded per plate (~ 3 X 10^5^ instead of ~ 5 X 10^5^).

### Statistical analyses

Statistical analyses were performed with Prism using a multiple t-test approach, assuming a uniform distribution with alpha set to 0.05 *(*P* < 0.05), **(*P* < 0.01), ***(*P* < 0.001), ****(*P* < 0.0001).

## Results

### The *M.tb* mutants deficient in more than one thiol have a growth defect in vitro

**Fig. 1:**
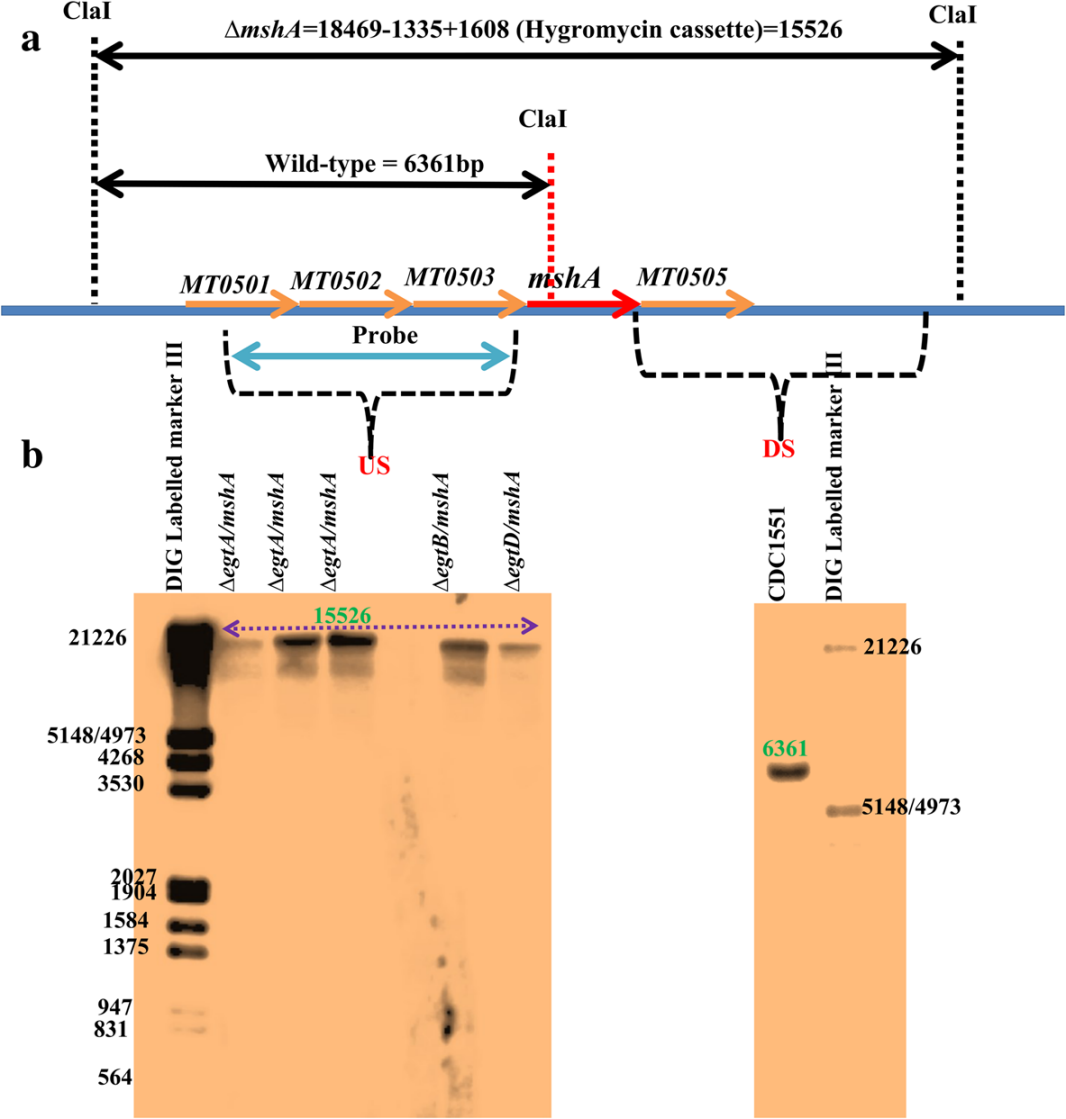
Southern blotting analysis of the double mutants. **a** Southern blotting design of *mshA* deletion. The gene *mshA* (1335 bp) was replaced by a hygromycin cassette (1608 bp) in the mutant. The restriction enzyme ClaI cuts within *mshA* but not within the hygromycin gene. It also cuts outside the cloned upstream (US) and downstream (DS) regions. Therefore, ClaI digestion of the wild-type genomic DNA and the mutant genomic DNA, yield distinct fragment sizes at the deleted region. These fragments are detected using a digoxigenin labelled DNA fragment (probe) that is able to hybridize to both fragments **b** Southern blotting results of the wild-type and mutants. Since the gene *mshA* is still intact in the wild-type strain, the 6361 bp fragment is detected. However, since *mshA* is replaced by the hygromycin cassette in the mutant, a bigger fragment is detected (15,526 bp)

The gene *mshA* was deleted in the previously generated *ΔegtA*, *ΔegtB*, *ΔegtD* single thiol-deficient mutants as previously described [18] (Fig. 1). This resulted in the loss of MSH production in the generated double mutants (Table 1). The *ΔegtA-mshA* mutant could not produce GGC, neither MSH, but did produce a low level of ERG, the *ΔegtB-mshA* could not produce ERG neither MSH but produced a significantly high level of GGC, and the *ΔegtD-mshA* mutant was also unable to produce ERG, neither MSH but produced a high level of GGC (Table 1). Complementation of the *ΔegtA-mshA* by inserting a copy of *egtA* at the attP site of the genome as previously described [18], restored the production of ERG and GGC in this strain (*ΔegtAc-mshA* in Table 1).

**Table 1:** Level of thiols in the generated double mutants pg/10^5^CFUs

	Wild-type	Δ*egtA-mshA*	Δ*egtB-mshA*	Δ*egtD-mshA*	Δ*egtAc-mshA*
IE_1_	111	10	< 5	< 5	486
EE_1_	33	15	< 5	< 5	303
IE_2_	296	48	< 5	< 5	184
EE_2_	93	44	< 5	< 5	118
IM_1_	41	< 5	< 5	< 5	< 5
IM_2_	12	< 5	< 5	< 5	< 5
IG_1_	1.7	< 5	112	24	25
IG_2_	0.6	< 5	91	23	21

*IE* intracellular ERG, *EE* extracellular ERG, *IM* intracellular MSH, *IG* intracellular gamma-glutamyclcysteine

**Fig. 2:**
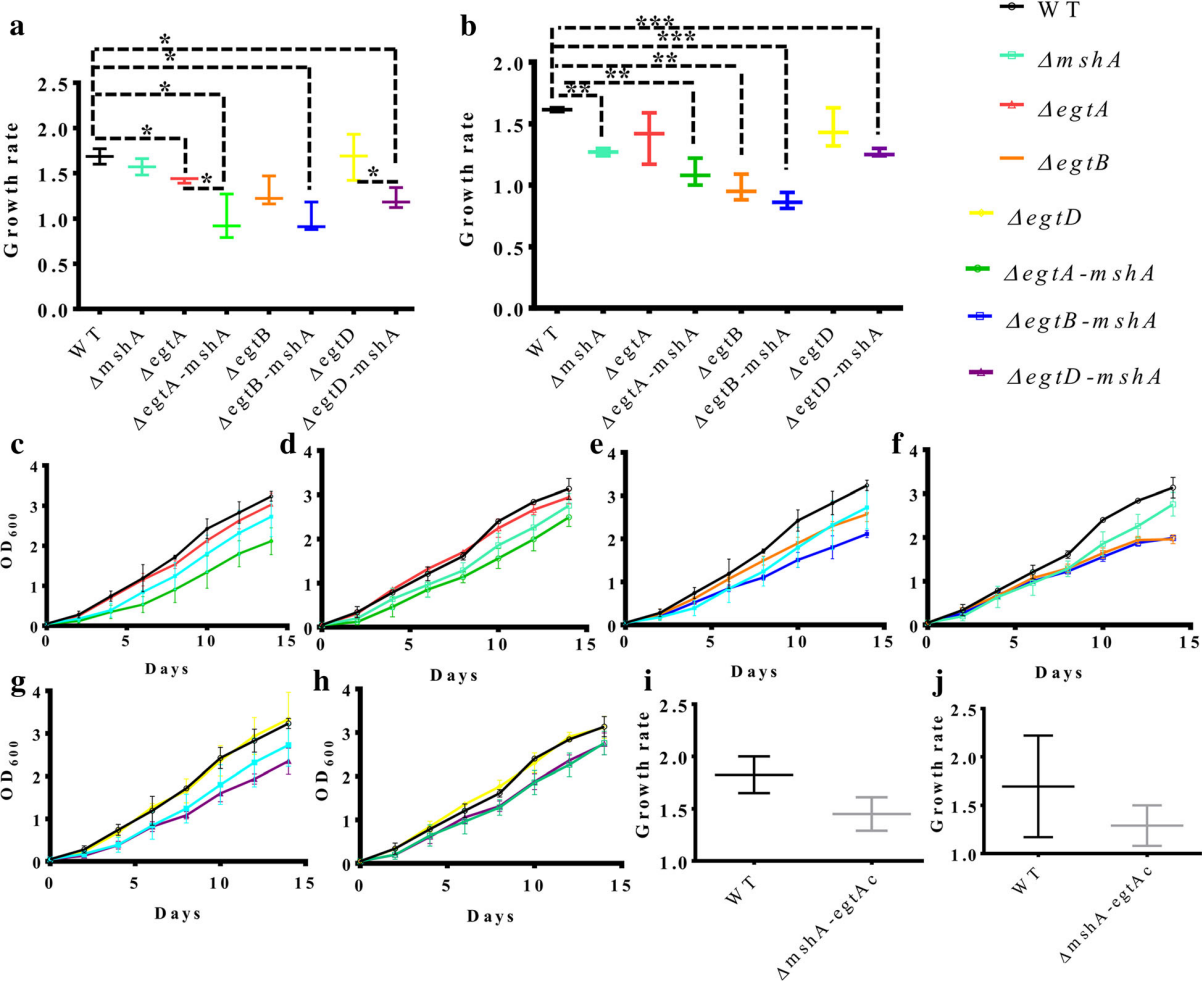
Growth curves of double mutants. **a** Growth rate of mutants in media supplemented with ADS, all double mutant display a growth defect relative to their parent single mutants and the wild-type (WT) strain. **b** Growth rate of mutants in media supplemented with OADC, all double mutant display a growth defect relative to the wild-type (WT) strain **c** Growth curves of the Δ*egtA-mshA* double mutant relative to its parent strains in media supplemented with ADS. **d** Growth curves of the Δ*egtA-mshA* double mutant relative to its parent strains in media supplemented with OADC **e** Growth curves of the Δ*egtB-mshA* double mutant relative to its parent strains in media supplemented with ADS **f** Growth curves of the Δ*egtB-mshA* double mutant relative to its parent strains in media supplemented with OADC. **g** Growth curves of the Δ*egtD-mshA* double mutant relative to its parent strains in media supplemented with ADS. **h** Growth curves of the Δ*egtD-mshA* double mutant relative to its parent strains in media supplemented with OADC. **i** Growth rate of the complemented strain of Δ*egtA-mshA* relative to the wild-type in media supplemented with ADS. **j** Growth rate of the complemented strain of Δ*egtA-mshA* relative to the wild-type in media supplemented with OADC. Data represent 2–3 experiments. Statistical analyses were performed with Prism using a multiple t-test approach, assuming a uniform distribution with alpha set to 0.05 *(*P* < 0.05), **(*P* < 0.01), ***(*P* < 0.001)

The growth profiles of the generated double mutants were evaluated in liquid cultures supplemented with either ADS or OADC. Growth fitness of the strains were evaluated by investigating their growth rate as previously described [26]. All double mutant had a growth defect in media supplemented with ADS or OADC relative to the wild-type strain (Fig. 2a and b). The growth defect of the Δ*egtA-mshA* mutant relative to its parent strains is more pronounced in the media supplemented with ADS (Fig. 2c) relative to media supplemented with OADC (Fig. 2d). This was not the case with the Δ*egtB-mshA* mutant (Fig. 2e and f), but was with the Δ*egtD-mshA* mutant (Fig.2g and h). The production of GGC and ERG is restored (Table 1) in the complemented strain of the Δ*egtA-mshA* mutant and analysis of the growth rate of this complemented strain relative to the wild-type reveals that it is not significantly (*P* > 0.05) different (Fig. 2i and j).

### The *M.tb* mutant, deficient in all three thiols, is the most sensitive to oxidative and nitrosative stress

**Fig. 3:**
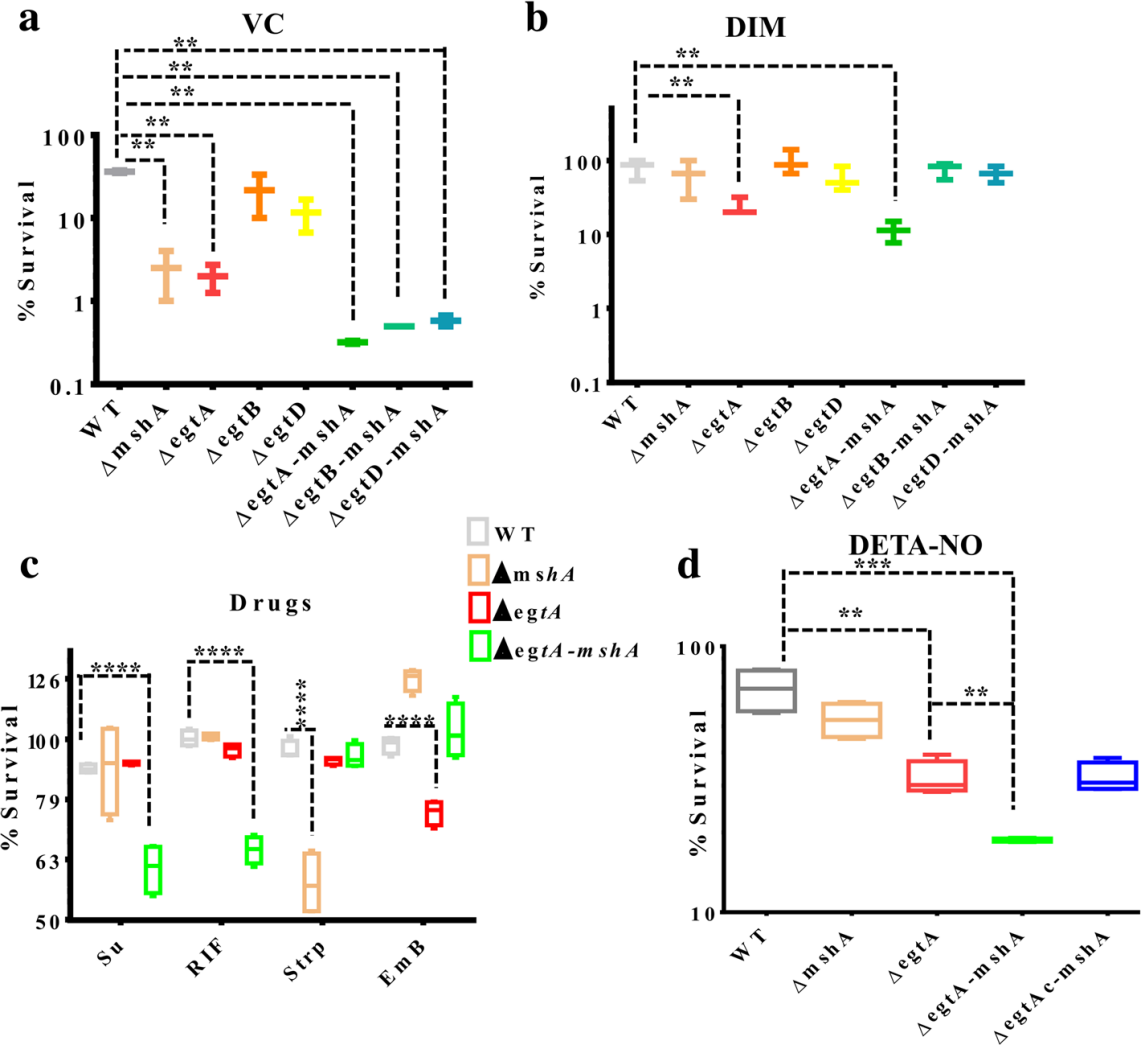
Susceptibility of the double mutants to oxidative and nitrosative stress. **a** Susceptibility of the mutants to vitamin C (VC) (5 mM for ~ 72 h). Every double mutant was more sensitive than its parent single mutant strains; however, Δ*egtA-mshA* appeared to be the most sensitive. Data are representative of two experiments. **b** Susceptibility of the mutants to diamide (DIM) (0.2 mM for ~ 72 h), Δ*egtA-mshA* is the most sensitive mutant. Data are representative of three independent experiments **c** Susceptibility of the mutants to sulfaguanidine (Su, 3.8 μg/ml), rifampicin (RIF, 0.0007 μg/ml), streptomycin (Strp, 0.16 μg/ml) and ethambutol (Emb, 0.6 μg/ml). Δ*egtA-mshA* is more sensitive than its parent strains to RIF and Su, while *ΔmshA* is sensitive to Strep and *ΔegtA* is sensitive to EmB. Data are representative of two experiments and two technical replicates for each experiment. **d** Susceptibility of the mutants to nitric oxide generated by diethylaminetriamine nitric oxide adduct (DETA-NO) (4 mM for 14 days). Even though, the single mutants display a sensitivity trend, Δ*egtA-mshA* is more sensitive than its parent strains. Data are representative of two experiments and two technical replicates for each experiment. Statistical analyses were performed with Prism using a multiple t-test approach, assuming a uniform distribution with alpha set to 0.05 *(*P* < 0.05), **(*P* < 0.01), ***(*P* < 0.001), ****(*P* < 0.0001)

Ferric ions (Fe^3+^) can be reduced by VC to ferrous ions (Fe^2+^) (Fe^3+^ + VC = Fe^2+^), which in turn can react with oxygen to yield superoxide (Fe^2+^ + O_2_ = O^**°**^_2_^−^ + Fe^3+^). The generated superoxide can be converted to hydrogen peroxide by dismutation (O^**°**^_2_^−^ + 2H^+^ = H_2_O_2_ + O_2_). Hydrogen peroxide can also react with ferrous ions to yield hydroxyl radicals (H_2_O_2_ + Fe^2+^ = Fe^3+^ + OH° + OH^−^) [27, 28]. As such, VC is able to generate oxidative stress. Previous studies indicated that the MSH-deficient *ΔmshA* mutant is sensitive to VC [29]. Therefore, in order to determine if the loss of more than one thiol would aggravate the sensitivity of *M.tb*, the double mutants with their respective parent single mutants were exposed to VC. While the *ΔegtA-mshA* mutant was the most sensitive strain (Fig. 3a), all three double mutants were more sensitive than their respective parent strains (Fig. 3a). Compounds such as DIM generate oxidative stress by oxidizing thiols [30, 31]. The mutant *ΔegtA-mshA* was the most sensitive double mutant to DIM (Fig. 3b). The susceptibility of this mutant to antibiotics known to generate oxidative stress such as rifampicin [32] (RIF) and sulfaguanidine [25] (Su) was investigated. Preliminary investigations at lethal concentrations revealed no significant difference between *ΔegtA-mshA* and its parent single mutant strains (*∆egtA* and ∆*mshA*) since they were already highly sensitive to these antibiotics as previously shown [11, 25]. However, sub-lethal concentrations of these drugs significantly inhibited the growth of the *ΔegtA-mshA* mutant (Fig. 3c). This was not observed with other tested antibiotics namely, streptomycin (Strp) and ethambutol (EmB) (Fig. 3c). It was previously indicated that the high level of MSH in the *∆egtA* mutant was able to protect it against a short exposure to a low concentration of nitric oxide. In addition, the high level of GGC in the *∆mshA* mutant was able to protect it against an extended exposure to a high concentration of nitric oxide [18]. In this study, the *ΔegtA-mshA* mutant (deficient in both MSH and GGC, Table 1) was more sensitive to nitric oxide stress generated by DETA-NO than the wild-type, the GGC-deficient *ΔegtA* and MSH-deficient *ΔmshA* single mutants (Fig. 3d).

### The *M.tb* mutant, deficient in all three thiols, has the most severe growth defect in macrophages

**Fig. 4:**
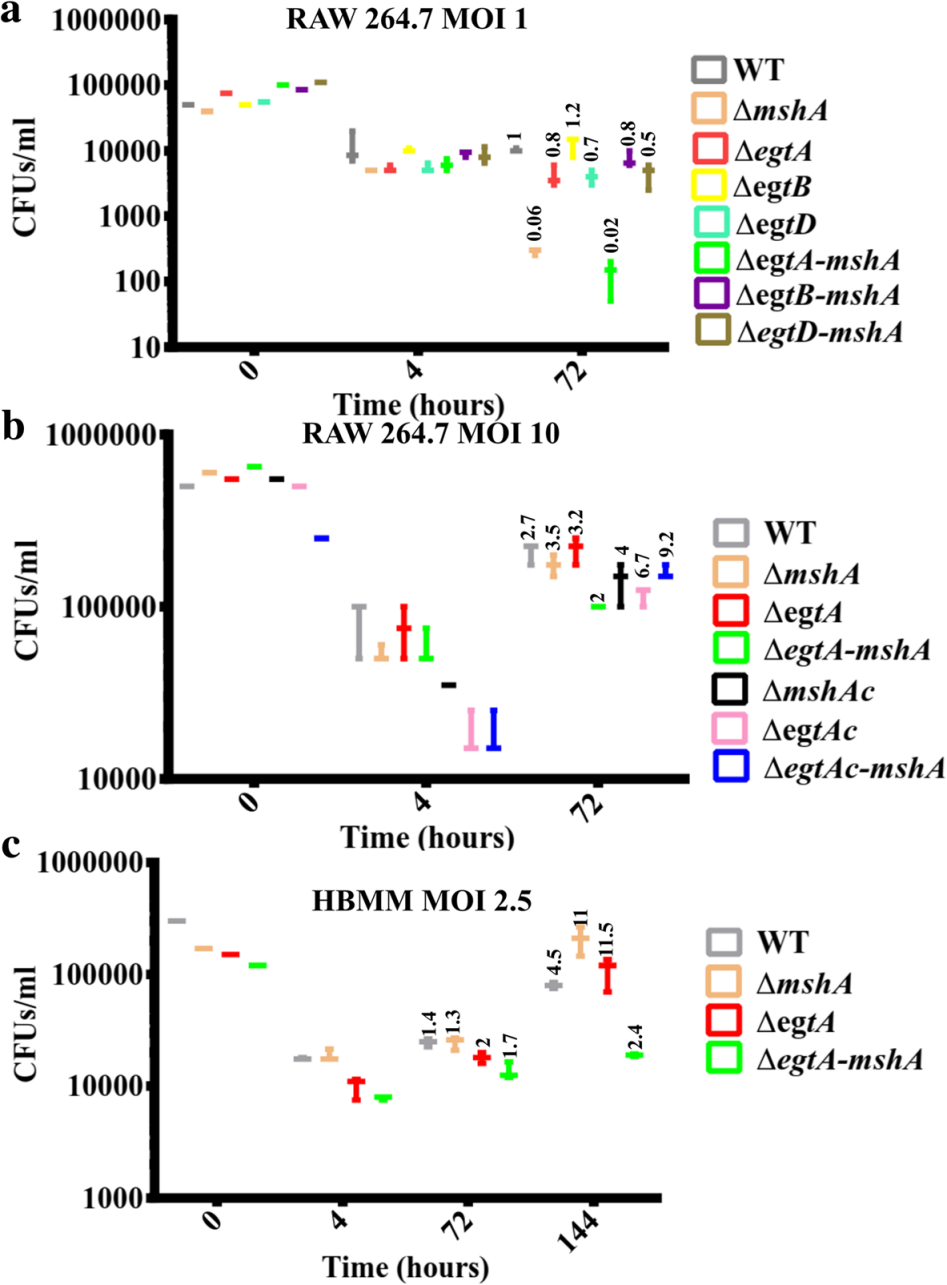
Survival of thiols-deficient mutants in macrophages. **a** Investigation of the survival of all double mutant relative to their parent strains in murine cell lines RAW264.7 at an MOI of~ 1:1. The Δ*egtA-mshA* double mutant was the most sensitive. **b** Further, investigation of its survival relative to its parent and complemented strains at an MOI of~ 10:1. The Δ*egtA-mshA* mutant was still the most sensitive. **c** This was also observed when human blood monocyte-derived macrophages (HBMM) were infected at an MOI of ~ 2.5:1 and 5:1 (data not shown), however at a later time point. The 0-h time-point represents the amount of mycobacteria added to the macrophages and the 4-h time point represents the amount of mycobacteria taken up by macrophages. For accuracy, the growth index (GI) was determined based on the amount of each ingested mycobacterial strain (4-h time point). The average GI indicated at the top of each whisker, was determined by dividing the CFUs obtained at the defined time point by the CFUs obtained at the 4-h time point (that indicates the uptake amount of each stain). Data are representative of three replicates

During TB infection, human macrophages are able to generate oxidative and nitrosative stress to destroy invading mycobacteria. *M. tuberculosis* is able to escape this defence mechanism to some extent [1]. It was previously shown that the thiol-deficient single mutants survived within the first few days and became sensitive after three days of infection in mouse macrophages at multiplicity of infection (MOI) ≥ 5:1 (mycobacteria: macrophages) [11, 33]. The ability of the double thiol-deficient mutants to survive within the first few days of infection of mouse macrophages was therefore investigated. The *ΔegtA-mshA* mutant was the most sensitive double mutant at MOI 1:1 (Fig. 4a). Investigation at a higher multiplicity of infection MOI 10:1 further confirms that the *ΔegtA-mshA* mutant was more sensitive than its parent single mutant strains during the first few days of infection (Fig. 4b). However, to ensure that these results could be related to humans treated with potential compounds inhibiting the biosynthesis of all three thiols, human blood monocyte-derived macrophages (HBMM) isolated from a healthy donor were infected with these mutants. As opposed to the observation in mouse macrophages (Fig. 4a and b), the *ΔegtA-mshA* mutant became more sensitive than its parent strains only after 3 days of infection at MOI 2.5:1 (Fig. 4c), a trend that was observed as well at a higher MOI (MOI 5:1, data not shown).

## Discussion

The ability of thiols to protect mycobacteria against oxidative and nitrosative stress is well documented [11, 17, 18, 33–35]. However, it was unclear if the fitness of *M.tb* would be more affected in case all thiols were depleted simultaneously. In this study, by generating a series of double mutants, it was shown that the loss of more than one thiol affects the growth rate of *M.tb*. In addition, the growth defect of the double mutants was more severe relative to their parent strains in the absence of catalase (media supplemented with ADS) (Fig. 2a, b, c, d, g, h). This suggests that catalase may facilitate the growth of these mutants by reducing hydrogen peroxide or peroxynitrite [36]. The growth defect phenotype was partially reversed in the complemented strain of the *ΔegtA-mshA* mutant (*ΔegtAc-mshA,* Fig. 2i and j). Since the production of ERG and GGC is restored in the complemented strain (Table 1), this suggests that all three thiols are required to ensure the optimal growth of *M.tb*.

On the other hand, though the Δ*egtB-mshA* and *ΔegtD-mshA* mutants were more sensitive than their parent single mutant strains to oxidative stress (Fig. 3a), the Δ*egtA-mshA* mutant was the most sensitive strain to oxidative and nitrosative stress (Fig. 3). In addition, it was sensitive to sub-lethal concentrations of RIF and Su, but not Strp neither EmB. Since the mode of action of Strp and EmB is not directly associated with the generation of oxidative stress [37], as is of the case for Su [25] and RIF [32], these results suggest that compounds targeting the biosynthesis of all three thiols would reduce the therapeutic dose of drugs that generate oxidative stress such as RIF and Su.

Though it was indicated that ERG is able to scavenge peroxyntrite [38], and decompose S-nitrosoglutathione [39], it is unclear if it is able to protect eukaryotic or prokaryotic cells against nitrosative stress. Preliminary investigations of the susceptibility of all three double mutants to nitrosative stress generated by DETA-NO revealed that the ∆*egtA-mshA* mutant, which still produced low levels of ERG (Table 1), was more sensitive than the Δ*egtB-mshA* and *ΔegtD-mshA* mutants which can’t produce ERG (Table 1) (data not shown). In addition, it was shown that chemical complementation of the sensitivity phenotype of the ∆*egtA* mutant to nitrosative stress generated by DETA-NO could be achieved with GGC but not ERG [18]. Therefore, it is less likely that the high sensitivity of the ∆*egtA-mshA* mutant to nitrosative stress relative to its parent strains (Fig. 3d) is associated with the depletion of ERG in this strain (Table 1). Nevertheless, according to results in Table 1, dealing with the anti-nitrosative roles of MSH [40] and GGC [18, 41], the sensitivity of the double mutant lacking both thiols (∆*egtA-mshA*) is more likely associated with the loss of both thiols (Table 1) in this strain.

Further investigations of the survival of these mutants revealed that the Δ*egtA-mshA* mutant had the most severe growth defect within macrophages (Fig. 4), indicating that the elevated GGC in the other double mutants (Table 1) may have protected them during the first three days of infection. The growth defect of the Δ*egtA-mshA* mutant within the mouse macrophages was observed during early infections (~ 3 days, Fig. 4a and b). On the other hand, the ERG-deficient single mutant strains were shown to display a growth defect within mouse macrophages after 4 days of infection [11, 33]. This suggests that the elevated levels of MSH and GGC in the ERG-deficient single mutants [18, 25] are not enough to protect them during a prolong exposure to ROS and RNS generated by macrophages (after 4 days) but are enough to protect them during the first few days of infection. Further explaining why, the Δ*egtA-mshA* mutant, deficient in all thiols was the most sensitive during the first few days of infection (Fig. 4a and b). However, this was not the case with human blood monocyte-derived macrophages (HBMM), as the Δ*egtA-mshA* mutant displayed the most severe sensitivity only after 3 days (Fig. 4c). This suggests agreement with previous studies that showed that mycobacteria are better adapted to survive and grow exponentially in the human primary macrophages than in mouse macrophages [42]. Probably, because mouse macrophages display increased iNOS (inducible nitric oxide synthase) activity and consequently increased production of RNS during early infections [43]. The sensitivity phenotype of the ∆*egtA-mshA* double mutant observed during the early time point in mouse macrophages (Fig. 4a and b) may therefore be due to this initial burst, which is not the case in HBMM where the growth defect was only observed at a later time point (6-day) (Fig. 4c). Therefore, thiols interplay to ensure an optimal protection of *M.tb* against nitrosative and oxidative stress. It is also worth noting that the Δ*egtA-mshA* mutant still produced a low level of ERG (Table 1), yet, it displayed the most severe sensitivity (Figs. 3 and 4) suggesting that the absolute simultaneous inhibition of all three thiols could be lethal to *M.tb*. However, this requires further investigations.

## Conclusions

In conclusion, thiols are able to protect *M.tb* against various cellular stresses and ensuring their fitness in vitro and within macrophages. However, to ensure the potential complete eradication of invading *M.tb* during infection, this study suggests that targeting the production of all three thiols simultaneously would be more efficient.

## Abbreviations

ADS: Albumin, dextrose, sodium chloride
CFU: Colony forming unit
CSPD: Disodium3-(4-methoxyspiro{l,2-dioxetane-3,2′-(5′-chloro)tricyclo[3.3.1.13,7]decan}-4-yl) phenyl phosphate
DETA-NO: Diethylaminetriamine nitric oxide adduct
DIG: digoxigenin
DIM: Diamide
DMEM: Dulbecco’s modified Eagle’s medium
EmB: Ethambutol
ERG: Ergothioneine
GGC: Gamma-glutamylcysteine
HBMM: Human blood monocyte-derived macrophages
Hr: hour
iNOS: Inducible nitric oxide synthase
*M.tb*: *Mycobacterium tuberculosis*
Min: minute
MOI: Multiplicity of infection
MSH: Mycothiol
OADC: Oleic acid, albumin, dextrose and catalase
PBMC: Peripheral blood mononuclear cells
PBS: Phosphate buffer saline
RIF: Rifampicin
RNS: Reactive nitrogen species.
ROS: Reactive oxygen species
RPMI 1640: Roswell Park Memorial Institute medium
RT: Room temperature
SDS: Sodium dodecyl sulphate
Strp: Streptomycin
Su: Sulfaguanidine.
TB: Tuberculosis
VC: Vitamin C
